# Early Blockade of CB1 Receptors Ameliorates Schizophrenia-like Alterations in the Neurodevelopmental MAM Model of Schizophrenia

**DOI:** 10.3390/biom12010108

**Published:** 2022-01-10

**Authors:** Tibor Stark, Fabio Arturo Iannotti, Serena Di Martino, Martina Di Bartolomeo, Jana Ruda-Kucerova, Fabiana Piscitelli, Carsten T. Wotjak, Claudio D’Addario, Filippo Drago, Vincenzo Di Marzo, Vincenzo Micale

**Affiliations:** 1 Department of Pharmacology, Faculty of Medicine, Masaryk University, 62500 Brno, Czech Republic; tibor_stark@psych.mpg.de (T.S.); jkucer@med.muni.cz (J.R.-K.); 2 Scientific Core Unit Neuroimaging, Max Planck Institute of Psychiatry, 80804 Munich, Germany; 3 Endocannabinoid Research Group, Institute of Biomolecular Chemistry, Consiglio Nazionale delle Ricerche, 80078 Pozzuoli, Italy; fabio.iannotti@icb.cnr.it (F.A.I.); fpiscitelli@icb.cnr.it (F.P.); vdimarzo@icb.cnr.it (V.D.M.); 4 Department of Biomedical and Biotechnological Sciences, Section of Pharmacology, School of Medicine, University of Catania, 95123 Catania, Italy; serena.dm.92@gmail.com (S.D.M.); f.drago@unict.it (F.D.); 5 Faculty of Bioscience and Technology for Food, Agriculture and Environment, University of Teramo, 64100 Teramo, Italy; mdibartolomeo@unite.it (M.D.B.); cdaddario@unite.it (C.D.); 6 Central Nervous System Diseases Research (CNSDR), Boehringer Ingelheim Pharma GmbH & Co KG, 88397 Biberach an der Riss, Germany; carsten.wotjak@boehringer-ingelheim.com; 7 Canada Excellence Research Chair on the Microbiome-Endocannabinoidome Axis in Metabolic Health, Faculty of Medicine and Faculty of Agricultural and Food Sciences, Centre de Recherche de l’Institut de Cardiologie et Pneumologie de l’Université et Institut sur la Nutrition et les Aliments Fonctionnels, Centre NUTRISS, Université Laval, Quebec City, QC G1V 4G5, Canada

**Keywords:** MAM model, schizophrenia, AM251, endocannabinoid system, 2-arachidonoylglycerol (2-AG), cannabinoid CB1 receptor

## Abstract

In agreement with the neurodevelopmental hypothesis of schizophrenia, prenatal exposure of Sprague-Dawley rats to the antimitotic agent methylazoxymethanol acetate (MAM) at gestational day 17 produces long-lasting behavioral alterations such as social withdrawal and cognitive impairment in adulthood, mimicking a schizophrenia-like phenotype. These abnormalities were preceded at neonatal age both by the delayed appearance of neonatal reflexes, an index of impaired brain maturation, and by higher 2-arachidonoylglycerol (2-AG) brain levels. Schizophrenia-like deficits were reversed by early treatment [from postnatal day (PND) 2 to PND 8] with the CB1 antagonist/inverse agonist AM251 (0.5 mg/kg/day). By contrast, early CB1 blockade affected the behavioral performance of control rats which was paralleled by enhanced 2-AG content in the prefrontal cortex (PFC). These results suggest that prenatal MAM insult leads to premorbid anomalies at neonatal age via altered tone of the endocannabinoid system, which may be considered as an early marker preceding the development of schizophrenia-like alterations in adulthood.

## 1. Introduction

There is an increasing amount of evidence suggesting that schizophrenia (SCZ) is, primarily, a developmental disease that becomes evident in adulthood [1]. Early neurodevelopmental anomalies may affect the typical brain maturational processes in the perinatal period causing a range of relatively benign changes that are markers of developmental disruption [2]. Emerging evidence indicates that preventive treatment with antipsychotics in the early phase of the disease could reduce the risk of progression to first-episode psychosis in patients [3,4] as well as the occurrence of behavioral and structural abnormalities in several experimental animal models [5]. Thus, the identification of susceptible individuals based on early life stress events and the presence of premorbid anomalies could give a chance of early pharmacological intervention in the prodromal period to prevent the transition to SCZ later in life [6]. In this regard, prenatal methylazoxymethanol acetate (MAM) exposure, which induces SCZ-relevant functional and neuropathological deficits at an adult age mimicking human conditions [7,8,9,10,11], resulted in a very useful experimental tool to assess the development of early postnatal deficits.

The endocannabinoid system (ECS) is mainly composed of: (i) the two endogenous ligands that are named anandamide (AEA) and 2-arachidonoylglycerol (2-AG), (ii) a large set of enzymes that are responsible for the biosynthesis and degradation of the two endocannabinoids, and (iii) at least the two G-protein-coupled receptors (the cannabinoid CB1 and CB2 receptors) for the endocannabinoids. The ECS, which recently emerged as a homeostatic regulator of synaptic neurotransmission that is involved in several behavioral responses [8,12,13,14,15,16], is also strongly implicated in the neurodevelopmental processes across the lifespan [17], starting already in fetal central nervous system (CNS) of rodents and humans [18]. More specifically, the levels of endogenous ligands as well as the expression of cannabinoid receptors fluctuate over different developmental stages in a temporal and region-specific manner [14], which, in turn, may drive cell proliferation, differentiation, and migration, as well synaptogenesis, positioning of cortical interneurons, and morphogenesis [19]. Alterations in ECS activity during the CNS development have been suggested to participate in the pathophysiology of SCZ [20,21]. However, still little is known whether prenatal MAM exposure could affect ECS signaling in the early postnatal days.

With this background in mind, in this study we have investigated the possible presence of subtle neurodevelopmental impairments during the early phase of postnatal life by assessing the development of neonatal physiological reflexes and nest-seeking behavior as indices of brain maturation [22]. Usually, altered development of neonatal milestones has been associated with dysfunctions of the CNS in human infants and in experimental models of neurodevelopmental disorders [23,24,25], dysfunctions that could be potentially associated with changes in ECS tone, and, in turn, be predictive of the development of SCZ-like deficits at adulthood.

## 2. Materials and Methods

### 2.1. Animals, MAM Model and Experimental Design

Methylazoxymethanol acetate (MAM; Midwest Research Institute, Kansas City, USA) administration was performed as previously described [26,27,28,29,30,31]. Briefly, timely pregnant female Sprague-Dawley rats were purchased from Charles River (Germany) at gestational day (GD) 13 and housed individually. They were randomly assigned to the MAM or to the control (CNT) experimental group. MAM (22 mg/kg, i.p.) or saline (1 mL/kg, i.p.) was administered on GD 17. The mothers were regularly weighed, and no differences were observed between the two experimental groups. Newborn litters that were found up to 5 pm were born on that day (postnatal day 0 = PND 0). At birth, no difference was found in the pregnancy length, total number of pups per litter, malformations, eye-opening time, or body weight. The male pups were weaned on PND 22 and housed in groups of 2–3 with littermates until adulthood when they were used for behavioral and neurochemical experiments, with food and water available *ad libitum* and under constant environmental conditions: relative humidity 50–60%, temperature 23 ± 1 °C, and 12-h light-dark cycle (lights on at 6 a.m.).

**Figure 1:**
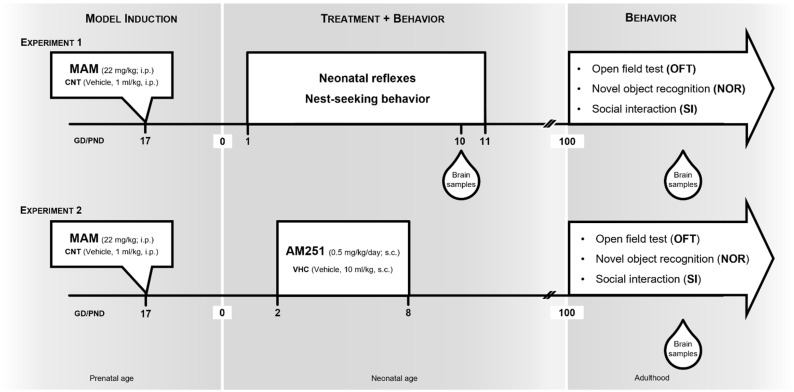
Experimental design that was used to investigate the effects of early pharmacological cannabinoid CB1 receptor blockade in MAM model of schizophrenia. Experiment 1: pregnant rats were exposed to methylazoxymethanol (MAM) acetate (22 mg/kg; i.p.) or saline (CNT: 1 mg/kg; i.p.) on gestational day (GD) 17. The resulting male offspring were subjected to behavioral tests at two different time points [neonatal age from postnatal day (PND) 1 to PND 11 and adulthood from PND 100], followed by neurochemical analysis. Experiment 2: pregnant rats were exposed to MAM acetate (22 mg/kg; i.p.) or saline (CNT: 1 mg/kg; i.p.) on GD 17. The resulting male offspring were treated from PND 2 to PND 8 with vehicle (VHC) or AM251 (0.5 mg/kg/day; s.c.). Behavioral and neurochemical analysis of the offspring were conducted at adulthood from PND 100.

A total of two different experiments were carried out. As described in Figure 1, in the first experiment, two groups of male MAM and CNT rats (*n* = 20 per group) were subjected to the evaluation of neonatal reflexes development from PND 1 to PND 11. On PND 11 they were subjected to nest-seeking behavior. From PND 100 they underwent behavioral assessment of locomotor activity, social, and cognitive performance.

In the second experiment, different groups of MAM and CNT rats were treated with the CB1 antagonist/inverse agonist AM251 (Sigma-Aldrich) that was dissolved in dimethylsulfoxide (DMSO), tween 80, and saline (1:1:8) and injected subcutaneously (s.c.) at the dose of 0.5 mg/kg/day from PND 2 to PND 8, based on previous results [32], or with the respective vehicle (VHC). As adults (from PND 100), these animals were submitted to the behavioral tests, which were conducted in battery with 3–4 days between two consecutive tests in the following order: the spontaneous locomotor activity in the open field test (OFT), the social activity in the social interaction (SI) test, and the cognitive performance in the novel object recognition (NOR) test [33].

After the completion of behavioral testing, the rats were decapitated in short ether anesthesia and their brains were collected. Based on previous evidence [9,14,27,28,34], the prefrontal cortex [(PFC) corresponding to an area that included the rostral pole of the brain, and delimited medially by the interhemispheric fissure, laterally by the corpus callosum, and caudally extended to AP +2.7 according to Paxinos and Watson (1998) [35] was dissected on ice by hand under microscopic control, within 2 min, immediately frozen in liquid nitrogen, and stored at −80 °C until analysis. All the rats showed normal body weight gain that was independent of both the prenatal (MAM or CNT) and of neonatal (AM251 or VHC) treatment. All the procedures were performed in accordance with EU Directive no. 2010/63/EU and approved by the Animal Care Committee of the Faculty of Medicine, Masaryk University, Czech Republic, and the Czech Governmental Animal Care Committee, in compliance with the Czech Animal Protection Act No. 246/1992.

### 2.2. Behavioral Testing

#### 2.2.1. Neonatal Reflexes

The development of neonatal behavior was studied by applying a battery of tests to assess the neonatal reflexes, which are considered reliable indices of neurological and behavioral development, as previously described [36,37,38]. A total of 20 males from each group (i.e., CNT and MAM, maximum of 2 pups per litter) were used for postnatal assessment of neurobehavioral development by a single examiner that was blind to the treatment conditions. Starting on PND 1, the newborn pups were weighed daily and observed for neonatal reflexes, until the “maximum appearance” was scored (i.e., 100% of the brood was found to exhibit the full repertoire of reflexes). The following reflexes were scored: (a) righting: the pup is capable of rapidly returning to its feet when placed on its back; (b) cliff aversion: the pup withdraws from the edge of a flat surface when its snout and forepaws are placed over a cliff that is 60 cm high; (c) forelimb placing: the pup places its forepaw up onto cardboard when it has been stroked with it against the dorsal surface of its paw; (d) forelimb grasping: the pup strongly grasps the barrel of the 16-gauge needle, 1.0-mm diameter, when it is touched against the palm of each forepaw; (e) bar holding: the pup holds itself on to a wooden stick, 2.0-mm diameter, for at least 5 s and (f) negative geotactic reaction: the pup turns its body 180° to face the upper side of the slope when placed head-down on an inclined surface (30°). Each pup was observed for maximum of 60 s.

#### 2.2.2. Nest-Seeking Behavior

On PND 11, 20 pups per treatment underwent determination of nest-seeking behavior, as previously described [24]. Briefly, the testing box consisted of a rectangular polycarbonate cage (40 × 20 × 18 cm) divided into three equal compartments by a permanent ink marker: a central arena and two side compartments, one side containing nest bedding from the test pup’s home cage and the same quantity of fresh clean bedding on the opposite side. Each pup was placed in the central arena; for nest seeking, crossing the line toward nest compartment with the forepaws and head was considered a positive entry. For nest exploration, crossing of the line plus sniffing and exploration of the nest were considered a positive score (cut-off time 60 s).

#### 2.2.3. Spontaneous Locomotor Activity

Exploratory activity was evaluated, as previously described with slight modifications [38,39,40,41,42,43,44]. The apparatus consisted of a cubic metal box (60 × 60 × 60 cm) that was moderately illuminated (80 lux). The floor of the box was divided into squares of equal size (15 × 15 cm). Briefly, each rat was individually placed in the center of the arena and allowed to explore for 30 min. During the observation period, the horizontal (the number of squares crossed with all paws) and the vertical (number of rearing episodes) exploratory activity was recorded and scored offline by two observers that were blind to the treatment groups. The arena was cleaned with 0.1% acetic acid and dried after each trial.

#### 2.2.4. Social Interaction (SI) Test

The test was carried out in a moderately illuminated room, as previously described [45,46,47]. A total of two unfamiliar rats, which had approximately the same weight and had received identical prenatal (MAM or saline) and postnatal (AM251 or VHC) treatment, were placed in opposite corners of a metal arena (120 lux, 60 × 60 × 60 cm) for 10 min. The arena was cleaned with 0.1% acetic acid and dried after each test. The social behaviors were defined as following, sniffing, grooming, nosing, and mounting. The whole testing phase was recorded and analyzed by two observers that were blind to the treatment groups. We scored the time spent in social behaviors and the number of interactions.

#### 2.2.5. Novel Object Recognition (NOR) Test

The experimental apparatus that was used for the NOR test was a metal arena (60 × 60 × 60 cm) that was placed in a moderately illuminated room (120 lux). Briefly, each rat was individually placed in the arena and allowed to explore two identical objects for 5 min (familiarization phase). After an inter-trial interval of 3 min, one of the two familiar objects was replaced by a novel object and rat was returned to the arena for the 5-min test phase. During the observation period, the time spent exploring the familiar object (Tf) and the new object (Tn) was videotaped and analyzed separately by two observers that were blind to the treatment groups, and the discrimination index (DI) was calculated as follows: (Tn − Tf)/(Tn + Tf). The arena and all the objects were cleaned with 0.1% acetic acid and dried after each trial [27,29,34].

### 2.3. Biochemical Methods

#### 2.3.1. Extraction, Purification and Quantification of Endocannabinoids and Endocannabinoids Related Compounds

The endocannabinoids anandamide (AEA) and 2-arachidonoylglycerol (2-AG) and endocannabinoid-related molecules N-palmitoylethanolamide (PEA) and N-oleoylethanolamide (OEA) were extracted from tissues and then purified and quantified as previously described [27,34]. First, the tissues were dounce-homogenized and extracted with chloroform/methanol/Tris-HCl 50 mM, pH 7.5 (2:1:1, *v*/*v*) containing internal deuterated standards for AEA, 2-AG, PEA, and OEA quantification by isotope dilution (5 pmol of [2H]8 AEA, 50 pmol of [2H]5 2-AG, [2H]4PEA, [2H]2 OEA (Cayman Chemicals, MI, USA). The lipid-containing organic phase was dried down, weighed, and pre-purified by open bed chromatography on silica gel. The fractions were obtained by eluting the column with 99:1, 90:10 and 50:50 (*v*/*v*) chloroform/methanol. The 90:10 fraction was used for AEA, 2-AG, PEA, and OEA quantification by liquid chromatography-atmospheric pressure chemical ionization-mass spectrometry (LC-APCI-MS) and using selected ion monitoring at M + 1 values for the four compounds and their deuterated homologues, as previously described [19,28].

#### 2.3.2. mRNA Extraction and Quantitative Real-Time Reverse Transcription-Polymerase Chain Reaction (qPCR)

Total RNA was isolated from native tissues by using TRI-Reagent (Sigma-Aldrich, Milan, Italy), according to the manufacturer’s instructions, reacted with DNase-I (1 U/mL; Sigma-Aldrich) for 15 min at room temperature, and followed by spectrophotometric quantification. The final preparation of RNA was considered DNA- and protein-free if the ratio between readings at 260/280 nm was ≥1.7. 1 μg of isolated mRNA was reverse transcribed by the use of iScript reverse transcriptase [Biorad (MI), Italy] in a 20 μL reaction volume with 1μL of iScript Reverse Transcriptase in 1X iScript Reaction Mix. The reaction mixes were incubated for 5 min at 25 °C, 20 min at 46 °C and 1 min at 95 °C. Quantitative real-time PCR was carried out in CFX384 real-time PCR detection system [Bio-Rad, Segrate (MI), Italy] by using a SYBR Green master mix kit [Bio-Rad, Segrate (MI)] with specific primers for the target genes [27,28,34]. The samples were amplified simultaneously in quadruplicate in one- assay run with a non-template control blank for each primer pair to control for contamination or primer-dimers formation, and the ct (cycle threshold) value for each experimental group was determined. Each PCR reaction (20 μL final volumes) was carried out with 100 ng of cDNA, 8μM of primers, and 1X SYBR green master mix. The housekeeping genes (the hypoxanthine-guanine phosphoribosyltransferase, hprt and/or ribosomal protein S16) were used as an internal control to normalize the ct values using the ΔΔCt method. The differences in mRNA levels between the groups are reported as 2−ΔΔCt (fold change) [27,28,34]. The primers that were used for PCR amplification are reported in Table S1.

#### 2.3.3. Western Blotting Analysis

The whole brain was dissected and washed twice in cold PBS (without Ca^2+^ and Mg^2+^, pH 7.4) and homogenized as previously described [27,34]. Lysates were then centrifuged for 15 min at 13,000× *g* at 4 °C, and the supernatants were transferred into clear tubes and quantified by DC Protein Assay (Bio-Rad, Milan, Italy). Subsequently the samples (60–80 µg of total protein) were boiled for 5 min in Laemmli SDS loading buffer and loaded on 8–10% SDS-polyacrylamide gel electrophoresis and then transferred to a PVDF membrane. The membranes were incubated overnight at 4 °C with the following antibodies: (a) rabbit polyclonal anti-CB1 Receptor Antibody (Y080037) Applied Biological Materials Inc. (CANADA); (b) rabbit polyclonal anti- DAGL α from Santa Cruz Biotechnology, Inc., Santa Cruz, CA, USA; (c) rabbit polyclonal anti-MAGL from Cayman Chemical (USA); and (d) mouse monoclonal anti-FAAH (WH0002166M7-100UG) from Sigma Aldrich MI Italy. The mouse monoclonal anti-tubulin clone B-5-1-2 (dilution 1:5000; Sigma–Aldrich, Milan, Italy) antibody was used to check for equal protein loading. Reactive bands were detected by chemiluminescence (ECL or ECL-plus; Perkin-Elmer, Waltham, MA, USA). The images were acquired and analyzed on a Chemi-Doc station with Quantity-one software (Bio-Rad) [27,34]. See Supplementary Figure S1 for the uncropped images of key immunoblot data that were presented in this study.

### 2.4. Statistical Analysis

The data were analyzed for statistical differences using GraphPad Prism version 9 (Graph-Pad Software, San Diego, CA, USA). Behavioral and molecular data were analyzed using two-way ANOVA [factor 1: model, factor 2: treatment] followed by *post-hoc* Fisher’s LSD for multiple comparisons, if appropriate. Unpaired *t*-test was used to analyze independent data (MAM vs. CNT) that was based on the result of the Shapiro-Wilk test of normality. The Fisher’s exact test was used for frequencies (comparison of reflex appearance percentage). Statistical significance was accepted at *p* < 0.05.

## 3. Results

### 3.1. Experiment 1: MAM Rats at Neonatal Age and Adulthood

**Figure 2:**
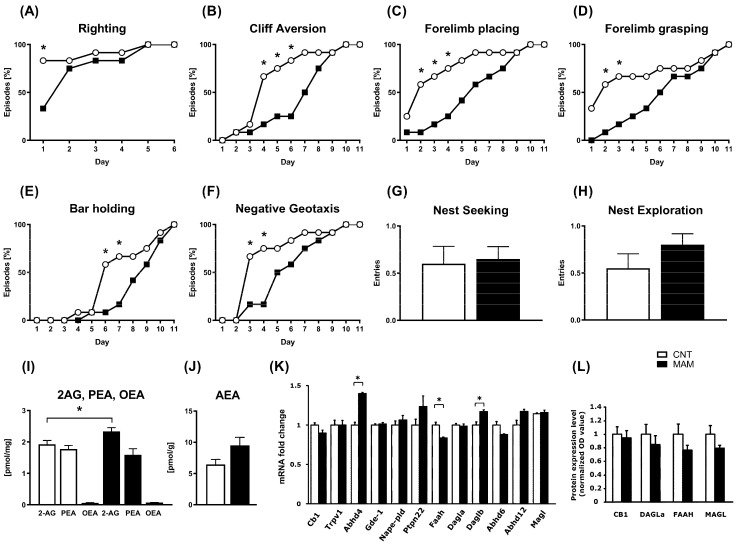
Effects of prenatal MAM exposure on neonatal behavior and endocannabinoid system (ECS) elements in rat pups. Data are presented as the mean values ±S.E.M.: (**A**–**F**) percentual cumulative appearance of each reflex on each day, per group of animals (*n* = 20), (**G**) the number of approaches towards maternal nest (nest seeking) and (**H**) nest exploration (*n* = 20/group), (**I**) 2-AG, PEA, OEA and (**J**) AEA levels, of (**K**) gene and (**L**) protein expression of ECS elements (receptors and metabolic enzymes; *n* = 3–5/group). * *p* < 0.05 vs. CNT.

The appearance rate of neonatal reflexes and the nest-seeking behavior in prenatally MAM-exposed rats and the respective controls (CNT) are shown in Figure 2. The percent appearance and the completion of neonatal reflexes had a significant delay in MAM rats. The Fisher’s exact test revealed several time points where the percentage of pups exhibiting righting (PND 1, *p* < 0.05; Figure 2A), cliff aversion (PND 4–6, *p* < 0.05; Figure 2B), forelimb placing (PND 2–4, *p* < 0.05; Figure 2C), forelimb grasping (PND 2–3, *p* < 0.05; Figure 2D), bar holding (PND 6–7, *p* < 0.05; Figure 2E), and negative geotaxis (PND 3–4, *p* < 0.05; Figure 2F) was significantly lower in the MAM group as compared to the CNT. No difference was found either in the number of approaches (*p* > 0.05; Figure 2G) or in the number of explorations (*p* > 0.05; Figure 2H) of maternal nests between the MAM and CNT groups.

In the whole brain of MAM rats, we found a significant increase of 2-AG content (t = 2.448, *p* < 0.05), but not of AEA (t = 1.909, *p* > 0.05), PEA (t = 0.8293, *p* > 0.05) or OEA (t = 1.125, *p* > 0.05) concentrations (Figure 2I,J) at PND 10. To extend these findings, we performed a wide transcriptomic analysis for all the genes that are known to encode for the large class of enzymes that are involved in the metabolism of the two major endocannabinoids (AEA and 2-AG), as well as of PEA and OEA. In fact, the two AEA-related compounds, PEA and OEA, can be also produced and degraded via the action of the same class of enzymes that control AEA tissue concentrations [48]. Using this approach, we found that, in the brain of MAM animals with respect to the CNT rats, the expression levels of the two endocannabinoid-responsive receptors, CB1 and TRPV1, were not significantly different (Figure 2K). In contrast, among the genes that are involved in the AEA metabolism, we found that α/β-hydrolase 4 (Abdh4; for the synthesis of AEA) and fatty acid amide hydrolase (FAAH; for the degradation of AEA) were slightly increased and reduced, respectively (Figure 2K). On the other hand, among the classes of genes regulating the metabolism of 2-AG, we did not find robust changes, but only a slight increase in diacylglycerol-lipase (Daglβ, involved in the biosynthesis of 2-AG) transcript levels (Figure 2K). Western blot analysis was then carried out to confirm the results of the transcriptomic analysis. No significant difference was detected in the protein expression of CB1 (t = 0.75413, *p* > 0.05), DAGLα (t = 0.48566, *p* > 0.05), FAAH (t = 0.26631, *p* > 0.05), or MAGL (t = 0.21111, *p* > 0.05) between the MAM and CNT groups (Figure 2L).

**Figure 3:**
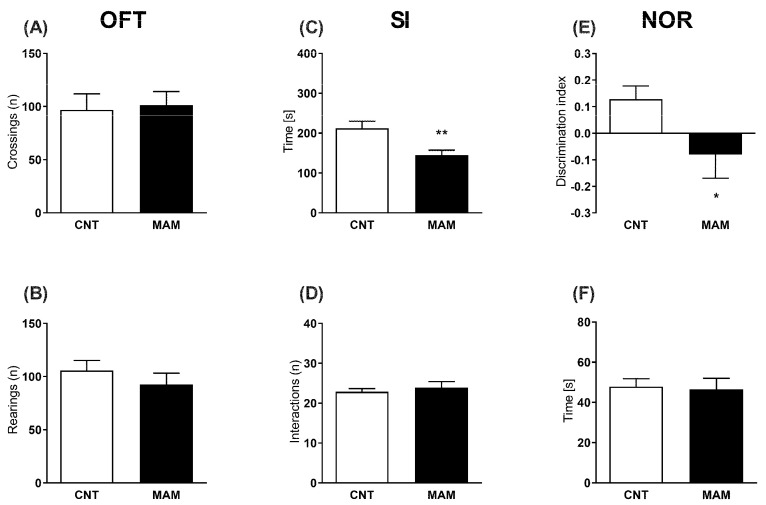
Effects of perinatal MAM exposure on the behavioral phenotype of rats that were tested (**A**,**B**) in the open field test (OFT), (**C**,**D**) in the social interaction (SI) test, and (**E**,**F**) in the novel object recognition (NOR) test at adulthood. Data are presented as means ±S.E.M. (*n* = 9–15). * *p* < 0.05 and ** *p* < 0.01 vs. CNT.

In adulthood, neither the spontaneous horizontal (number of entries, t = 0.2292, *p* > 0.05) nor the vertical (number of rearings, t = 0.9060, *p* > 0.05) locomotor activity in a novel environment was modified by prenatal MAM exposure (Figure 3A,B). By contrast, prenatal MAM-exposed rats showed a reduced time of interaction as an index of social withdrawal in the SIT (t = 3.164, *p* < 0.01 vs. CNT; Figure 3C). No difference was found in the number of interactions between the MAM and CNT groups (t = 0.5611, *p* > 0.05; Figure 3D). The MAM rats also showed a cognitive deficit in the NOR as described by a lower discrimination index (t = 2.108, *p* < 0.01 vs. CNT; Figure 3E). No difference was found in the total exploration time between the groups (t = 0.2089, *p* > 0.05; Figure 3F).

### 3.2. Experiment 2: Effects of Early Blockade of CB1 Receptor in MAM Rats at Adulthood

**Figure 4:**
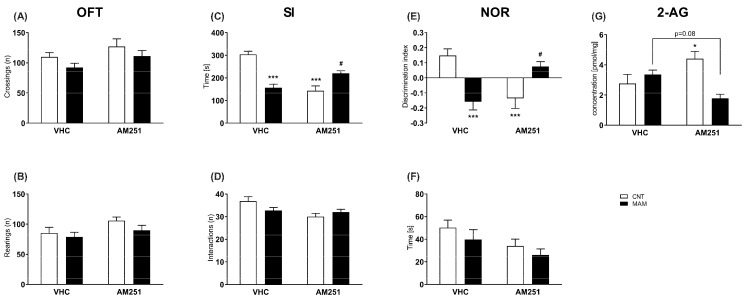
Effects of early pharmacological blockade of cannabinoid CB1 receptor on MAM rats at adulthood. Data are presented as means ±S.E.M (*n* = 7–12) of (**A**,**B**) locomotor activity in the OFT, (**C**,**D**) social behavior in the SI, (**E**,**F**) cognitive performance in the NOR, and of (**G**) (*n* = 3–5) 2-AG content in the PFC. * *p* < 0.05 and *** *p* < 0.01vs. CNT/VHC; # *p* < 0.05 vs. MAM/VHC.

Neither prenatal MAM exposure nor AM251 treatment affected the spontaneous horizontal (number of crossings: two-way ANOVA, factor model: F(1, 31) = 3.443, *p* > 0.05; factor treatment: F(1, 31) = 4.067, *p* > 0.05; factor model x treatment interaction: F(1, 31) = 0.01190, *p* > 0.05) or vertical locomotor activity (number of rearings: two-way ANOVA, factor model: F(1, 31) = 1.848, *p* > 0.05; factor treatment: F(1, 31) = 3.522, *p* > 0.05; factor model x treatment interaction: F(1, 31) = 0.3497, *p* > 0.05; Figure 4A,B).

As described in Figure 4C, the MAM/VHC group spent less time in social interaction (two-way ANOVA, factor model: F(1, 30) = 4.288, *p* < 0.05; factor treatment: F(1, 30) = 8.149, *p* < 0.01; factor model x treatment interaction: F(1, 30) = 44.50, *p* < 0.01) as compared to the CNT/VHC rats (*p* < 0.001), indicating impaired social behavior. Early treatment with AM251 improved the social performance in the MAM group as compared to the MAM/VHC group (*p* < 0.05). However, in the CNT group, AM251 reduced social activity (*p* < 0.001). Neither prenatal MAM exposure nor AM251 treatment (two-way ANOVA, factor model: F(1, 30) = 0.2606, *p* > 0.05; factor treatment: F (1, 30) = 3.665, *p* > 0.05; model x treatment interaction: F(1, 30) = 2.478, *p* > 0.05) affected the number of interactions as an index of locomotor activity (Figure 4D).

In the NOR test, prenatal MAM exposure affected the recognition memory as described by a significant reduction (*p* < 0.001) in the discrimination index (two-way ANOVA, factor model: F(1, 37) = 0.9242, *p* > 0.05; factor treatment: F(1, 37) = 0.3193, *p* > 0.05; model x treatment interaction: F(1, 37) = 18.89, *p* < 0.001) during the test phase, which was reversed by AM251 (*p* < 0.05). In the CNT group, AM251 treatment impaired the cognitive performance (*p* < 0.001) as compared to CNT/VHC group (Figure 4E). No difference was found in the total exploration time among the groups (two-way ANOVA, factor model: F(1, 37) = 0.02402, *p* > 0.05; factor treatment: F(1, 37) = 3.995, *p* > 0.05; model x treatment interaction: F(1, 37) = 1.521, *p* > 0.05; Figure 4F).

At the molecular level we found that prenatal MAM did not affect 2-AG content (*p* > 0.05 vs. CNT/VHC) in the PFC of adult rats (two-way ANOVA, factor model: F(1, 13) = 3.597, *p* > 0.05; factor treatment: F(1, 13) = 0.005, *p* > 0.05; model x treatment interaction: F(1, 13) = 9.055, *p*<0.05). Based on the significant interaction between the model and treatment, post-hoc analysis revealed that in the CNT group, the neonatal CB1 blockade significantly enhanced 2-AG content in the PFC of rats (*p* < 0.001 vs. CNT/VHC group; Figure 4G), while it induced a trend of decreased content in the MAM animals (*p* = 0.08 vs. MAM/VHC group; Figure 4G).

## 4. Discussion

We showed that gestational MAM exposure delays the onset of neonatal reflexes, which are considered a predictive factor for CNS dysfunctions both in human infants and in preclinical models of neurodevelopmental disorders (i.e., schizophrenia or autism) [24,25,34]. Neurological reflexes are involuntary and repetitive movements showing the maturity of the brain stem, spinal cord, and higher cortical networks, which in turn are characterized by myelination and synaptogenesis processes [49]. Alterations in the CNS evolution can affect the brain development, resulting in abnormal cortical wiring, functioning, and myelination, which could play a role in altered developmental milestones. Neonatal sensory-motor reflex development (e.g., righting, negative geotaxis, cliff aversion) requires coordination among the vestibular system, muscles and spine; thus, a delay in their appearance could also indicate retarded muscular development and growth, as previously suggested [49]. Prenatal MAM insult failed to affect nest-seeking behavior [24,50], a simple form of early-life learning that integrates the olfactory identification of relevant cues (i.e., feeding mother), goal-oriented locomotion, spatial navigation, sensory, associative and discriminative capabilities [51]. Indeed, the effects of prenatal MAM insult can be detected before puberty, in line with population-based studies and supporting the hypothesis that the clinical course of SCZ may develop at stages with subtle deficits during early childhood (i.e., abnormal milestones, neuromotor and cognitive impairments) recognized as premorbid phase [52,53]. At the molecular level, at PND 10 (when all reflexes are fully expressed) we detected elevated 2-AG brain levels in the MAM pups, partially due to the increased expression of the biosynthetic enzyme DAGL-β and concomitant slight reduction of the gene encoding for the degradative enzyme ABDH4 that was in agreement with previous results in different SCZ animal models [54,55]. However, given that DAGL-β in early postnatal 2-AG biosynthesis is progressively replaced by DAGL-α in the developing brain and the regulation of both is temporally and spatially diverse [56], it is possible that other mechanisms also contribute to increased 2-AG levels in the brains of MAM pups. 2-AG can suppress glutamate release by activating the cannabinoid CB1 receptors in presynaptic glutamatergic axon terminals [57,58]. Thus, the neonatal induction of brain 2-AG signaling by prenatal MAM exposure could reduce glutamatergic neurotransmission (in terms of NMDA receptor hypofunction, reduced NMDA and mGLU5 receptor expression or altered epigenetic regulation of NR2B subunit gene expression) which is a typical hallmark both in SCZ subjects [59] and in the MAM model [60,61,62,63]. We cannot also exclude that impaired dopaminergic [64] and/or GABAergic signaling [65] as well as altered neuronal density may also play a role in the abnormal early reflexes [66]. Overall, our data support the idea that altered ECS signaling at neonatal age could negatively affect the maturational processes within the CNS leading to abnormal neurotransmission, which could, in turn, underlie the social and cognitive deficits in adulthood (Figure 3). Further studies for assessing neuronal maturation markers such as synaptophysin, neuronal nuclear antigen, neurofilaments protein, or calretinin are needed [67]. It is important to keep in mind that reflexes mirror a much more complicated network, while we provided a general correlation here. Nevertheless, these reflexes represent a quick and easy way to assess neurological development at very young age when more complex behavioral testing is not feasible.

As expected [7,8,11,27], we observed that MAM administration at GD 17 induces social and cognitive deficits at adulthood, as suggested by a lower discrimination ratio in the NOR and a reduced time of interaction in the SI. These are usually recognized as indices of altered recognition memory and social deficits, respectively, which, in turn, are considered reminiscent of SCZ symptoms [68]. Furthermore, the total object exploration time was similar among the treatment groups, suggesting that the lower discrimination ratio that was observed in the prenatal MAM-exposed rats was not due to the lack of object exploration (i.e., impaired locomotor activity), but to the lack of discrimination between the novel and the familiar objects. Interestingly, the social withdrawal, a behavioral correlate of negative symptoms of SCZ, was also not related to the motor activity of the MAM rats since no difference was found in the number of interactions. Indeed, locomotor activity paradigms are used as an internal control to assess the possible unspecific stimulant effects which may confound the interpretation of the behavioral parameters. The locomotor activity was not altered in the MAM animals in our experimental conditions which is in line with previous results [9,27,29]; thus, our study reinforces the original findings that cognitive impairment and social withdrawal are robust phenotypes in the MAM model. However, further investigations are needed to assess the effects of early cannabinoid CB1 blockade on additional cognitive and social domains which are impaired in SCZ.

In agreement with previous evidence showing that the ECS is overactive during SCZ development, we propose the modulation of EC signaling, and, in particular, the CB1 receptors as therapeutic targets for the treatment of schizoaffective disorders [20]. To support this hypothesis, we report here for the first time that a neonatal blockade of cannabinoid CB1 receptor counteracted the development of recognition memory impairment that was induced by prenatal MAM exposure, which at least seems to be mediated by higher 2-AG brain content at a neonatal age. Our results are in line with previous data showing that cannabinoid CB1 blockade could ameliorate the cognitive performance in animal models of SCZ [55,69,70]. The cannabinoid CB1 receptor is present at very high levels on inhibitory (GABAergic interneurons) and to a lesser extent on excitatory (glutamatergic) terminals [71] as well as on dopamine D1-expressing neurons, playing a modulatory role on different emotional behaviors such as social and cognitive activity [33,45,72]. Indeed, we cannot exclude that AM251 effect could be mediated by specific neuronal subpopulations as well as the involvement of several neurotransmitter systems such as the cholinergic transmission, which is enhanced by cannabinoid CB1 receptor blockade in PFC [73,74]. In addition to its beneficial effect on cognitive performance, repeated neonatal AM251 treatment antagonized the social withdrawal in MAM rats, which could be also mediated by higher neonatal 2-AG content. This finding is also in line with previous results, showing the efficacy of CB1 antagonists to ameliorate social withdrawal in SCZ animal models [20,27], which, in turn, could be also due to the normalization of 2-AG content during brain development. Interestingly, we observed that AM251, *per se*, elicited detrimental effects in the control animals (see Figure 3B,C) and these were again accompanied by enhanced 2-AG concentrations in the PFC, a key region for the integration of cognitive and negative signs of SCZ [68,75,76,77]. This finding is in agreement with our hypothesis that elevated 2-AG levels and CB1 tone in the PFC of adult MAM rats may contribute to SCZ signs in this model. The mechanisms underlying the enhanced 2-AG levels in CNT rats remain undetermined and may be due to changes in the glutamatergic and/or dopaminergic signaling as previously suggested [69]. These results further provide evidence that cannabinoids trigger different behavioral responses in distinct experimental groups (CNT vs. MAM) that is consistent with previous reports in individuals with SCZ vs. healthy subjects [78,79] and in laboratory-based studies [27,69,80]. A potential gender-related effect was described in SCZ development [81], in the expression and functionality of ECS elements (i.e., cannabinoid CB1 receptor) [82], as well as in the effects of cannabinoid exposure [83]. Thus, further studies will be required to clarify the behavioral and molecular responses of female offspring both to prenatal MAM exposure and to the early pharmacological CB1 blockade.

## 5. Conclusions

We showed here that prenatal MAM exposure induces neonatal behavioral and molecular alterations involving EC signaling as associative and potentially predictive signs of SCZ-like deficits at adulthood. Based on our results, the early reduction of EC signaling overactivity may thus circumvent the ECS dysregulation that may contribute to the emergence of a SCZ-like phenotype in adulthood. Further studies are necessary to assess the effects both of early CB1 blockade on different neurotransmitter systems (i.e., dopaminergic, glutamatergic, GABAergic) and of novel compounds acting on cannabinoid CB1 receptors (i.e., the peripherally restricted CB1 inverse agonist/antagonist or the cannabinoid CB1 neutral antagonists), which seem to display a better safety profile as compared to the cannabinoid CB1 inverse agonists [84,85]. Based on our present results, selective inhibitors of DAGL-β [86] may also counteract the MAM-induced SCZ signs in rats and should therefore be tested in future studies.

## Data Availability

Data can be provided from the corresponding author upon reasonable request.

## Supplementary Materials

The following supporting information can be downloaded at: https://www.mdpi.com/article/10.3390/biom12010108/s1, Table S1: List of primer sequences used for quantitative real-time RT-PCR analysis. Figure S1: The uncropped images of key immunoblot data.
