# Supplementary material for: Early Blockade of CB1 Receptors Ameliorates Schizophrenia-like Alterations in the Neurodevelopmental MAM Model of Schizophrenia
Source: Biomolecules. 2022 Jan 10;12(1):108. doi: 10.3390/biom12010108 (PMC8773886; doi:10.3390/biom12010108)
Supplement: Supplementary file 1 [file biomolecules-12-00108-s001.zip › supple-Table S1.pdf]

Table S1.
List of primer sequences used for quantitative real-time RT-PCR analysis.

**Gene name:**	**Forward sequence (5’- 3’)**	**Reverse sequence (5’- 3’)**
Abhd4	TCTGGCGTCAAGCGGAGGGA	ACGCCACCCCCAAAGCCATG
Abdh6	AGCGTCTGCTCCCATCCCCA	TGGCTTGCCAGTGGCGTGAA
Abdh12	CAGGCGTGCGGTCGAAACCA	TCAAGCTGCAGTCGGCGTCC
Cnr1	CTGAGGGTTCCCTCCCGGCA	TGCTGGGACCAACGGGGAGT
Daglα	GGCCGCACCTTCGTCAAGCT	ATCCAGCACCGCATTGCGCT
Daglβ	AGACCCGGGTGCAATGCTGC	GCCCTGGTGTGTGGGTCACG
Faah	GGCAGAGCCACAGGGGCTATCA	TGGGGCTACAGTGCACAGCG
Gde1	GCAGCCCCTTCAACGCCTGT	GATGGCCGCCAGCGTGTTCT
Napepld	AGGCTGGCCTACGAATCACGT	ATGGTACACGGGGGACGGCG
Ptpn22	TGGTCGTGGGAGAGCCGCTT	GGGCCACTTTTTGCGCCTGC
Trpv1	AGACATCAGCGCCCGGGACT	CCAGCTTCAGCGTGGGGTGG
Hprt	ACAGGCCAGACTTTGTTGGATTTGA	AGGCTGCCTACAGGCTCATAGTG
S16	TCCGCTGCAGTCCGTTCAAGTCTT	GCCAAACTTCTTGGATTCGCAGCG
