# Supplementary figures and images for: Early Blockade of CB1 Receptors Ameliorates Schizophrenia-like Alterations in the Neurodevelopmental MAM Model of Schizophrenia
Source: Biomolecules. 2022 Jan 10;12(1):108. doi: 10.3390/biom12010108 (PMC8773886; doi:10.3390/biom12010108)

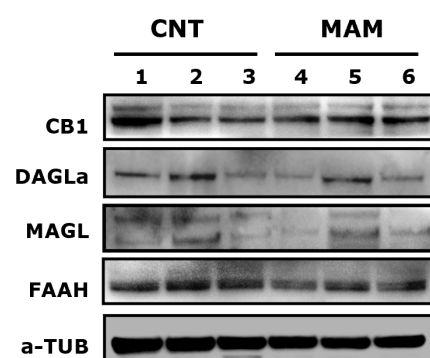

**Figure S1.** The uncropped images of key immunoblot data.

Supplement: Supplementary file 1 [file biomolecules-12-00108-s001.zip › supple-Figure S1.pdf]
